# A Systematic Literature Review for Evidence of *Aphanizomenon flos-aquae* Toxigenicity in Recreational Waters and Toxicity of Dietary Supplements: 2000–2017

**DOI:** 10.3390/toxins10070254

**Published:** 2018-06-21

**Authors:** Amber Lyon-Colbert, Shelley Su, Curtis Cude

**Affiliations:** 1 School of Biological and Population Health Science, Oregon State University, Corvallis, OR 97331, USA; lyoncola@oregonstate.edu (A.L.-C.); shelley.su@oregonstate.edu (S.S.); 2 Oregon Health Authority, Public Health Division, Portland, OR 97232, USA

**Keywords:** *Aphanizomenon flos-aquae*, blue-green algae supplements, cyanotoxins, microcystin, cylindrospermopsin, saxitoxin

## Abstract

Previous studies of recreational waters and blue-green algae supplements (BGAS) demonstrated co-occurrence of *Aphanizomenon flos-aquae* (AFA) and cyanotoxins, presenting exposure risk. The authors conducted a systematic literature review using a GRADE PRISMA-p 27-item checklist to assess the evidence for toxigenicity of AFA in both fresh waters and BGAS. Studies have shown AFA can produce significant levels of cylindrospermopsin and saxitoxin in fresh waters. Toxicity studies evaluating AFA-based BGAS found some products carried the *mcyE* gene and tested positive for microcystins at levels ≤ 1 μg microcystin (MC)-LR equivalents/g dry weight. Further analysis discovered BGAS samples had cyanotoxins levels exceeding tolerable daily intake values. There is evidence that *Aphanizomenon* spp. are toxin producers and AFA has toxigenic genes such as *mcyE* that could lead to the production of MC under the right environmental conditions. Regardless of this ability, AFA commonly co-occur with known MC producers, which may contaminate BGAS. Toxin production by cyanobacteria is a health concern for both recreational water users and BGAS consumers. Recommendations include: limit harvesting of AFA to months when toxicity is lowest, include AFA in cell counts during visible blooms, and properly identify cyanobacteria species using 16S rRNA methods when toxicity levels are higher than advisory levels.

## 1. Introduction

Cyanobacteria, also known as blue-green algae, are photosynthetic bacteria that occur in many fresh and salt water environments around the world. Some cyanobacteria species are toxigenic; they have the potential to produce toxins that can harm people, pets and wildlife. *Aphanizomenon flos-aquae* (AFA) is naturally present in fresh-water sources and has been harvested as dietary blue-green algae supplements (BGAS) in the U.S. since the 1980s [1,2]. AFA is one of the most common species of cyanobacteria collected from the natural environment. In freshwater environments, several cyanobacteria species including *Aphanizomenon* spp. form the most common and noxious type of harmful cyanobacteria blooms (CyanoHABs), which have potentially dire consequences for environmental and human health [3,4]. The potential danger that exposure to cyanotoxins presents is widely known [2,5], and has been estimated to cause between 50,000 to 500,000 human intoxications per year from consumption of finfish and shellfish [6]. While the liver is the primary target organ of microcystins (MCs), other organs can be affected as well [7]. Previous studies have shown effects on the heart, nervous system, kidneys, and GI tract [8]. Very few earlier studies addressed the variability of toxin content in BGAS, although this knowledge would have been important for risk assessments. Long-term consumption of BGAS containing harmful cyanotoxins is cause for public health concerns as they are widely available, labeled as safe products and promoted as beneficial for health. In addition, a plethora of fatalities and severe illness/injury have been recorded worldwide in pets, livestock, birds, wildlife and fish [9,10,11,12]. Most cyanotoxin poisonings have occurred when animals drink cyanobacterial-laden freshwater, but other aquatic animals, including fish and shellfish, are also affected [13]. These effects include diarrheal illness, acute liver damage, and even more serious and potentially fatal neurotoxic outcomes. 

Cyanotoxin guidelines were established by the World Health Organization (WHO) in 1999 [5]. Work is ongoing to determine appropriate lethal dose for 50 percent of the test population (LD_50_) and tolerable daily intake (TDI), or thresholds for daily reference doses (RfD). Under the Clean Water Act (CWA) 304(a) the Environmental Protection Agency (EPA) recommended a threshold for RfD concentrations of cyanotoxins to protect human health while swimming or participating in other recreational activities in and on the water [14]. States can adopt the EPA criteria into their water quality standards and use these same threshold values as the basis of swimming advisories for public notification purposes at beaches [14]. States may also use the proposed EPA RfD values when adopting new or revised water quality standards (WQS). If adopted as WQS and approved by the EPA under CWA 303(c), the WQS could be used for all CWA purposes. The EPA has also noted that currently, “available data are insufficient to develop quantitative recreational values for cyanobacterial cell density related to health outcomes” [14]. The epidemiologic associations between cell density and specific health outcomes described in the literature are not consistent [14]. In addition, epidemiologic studies addressing species and strains of cyanobacteria and cell densities associated with significant health effects do not provide sufficient information to determine a consistent association between cyanobacteria cell counts and adverse health outcomes [14]. 

Because of incidents attributed to toxic CyanoHABs, the WHO and the EPA recommended that risk assessment plans and safety levels include cyanobacteria as an environmental parameter to be monitored and assessed. Swimmers can involuntarily swallow water while swimming, and harm from ingestion of recreational water is comparable to that of drinking water with the same toxin content [5]. For recreational water users with whole-body contact, a swimmer can expect to ingest 100–200 mL of water in one session, with sailboard riders and water skiers ingesting more depending upon age and length of exposure [5]. AFA is reported to be capable of producing anatoxin-a (ANTX), cylindrospermopsin (CYN), microcystins (MCs), and the paralytic shellfish poison toxins (PSP toxins), saxitoxins (STXs), though there have been conflicting reports and varying advisory levels among scientists and health departments [5,13,14,15]. In addition, laboratory studies with pure strains of cyanobacteria have found that environmental factors can induce changes in toxicity or toxin concentration [5]. The factors that control the growth and toxin content of individual strains are unknown, but the regulation of cyanotoxin production is a critical area for further study and understanding [5]. Most existing studies say that cyanobacteria produce the most toxins under favorable environmental conditions for growth, such as temperature, light, and pH levels [4,5]. Although different cyanobacteria species have differing light and temperature requirements, all cyanobacteria strains produce the most toxins when grown under optimal light conditions [5]. Traditionally we have seen seasonal patterns of CyanoHABs with the majority reported during the warmer summer months [14]. This may lead to recommendations for seasonal harvesting of AFA when MC concentration would be lowest, and for increased testing and warnings for recreational use and seafood consumption during times of high MC levels. 

The aim of this systematic review is to determine the strength of evidence for the toxigenicity of *Aphanizomenon flos-aquae* in order to evaluate the risks posed to recreational water users and dietary BGAS consumers. 

## 2. Results

### 2.1. Toxigenicity

Some species of cyanobacteria are considered toxigenic, meaning they carry the genes responsible for producing various toxins that are classified by mode of action into hepatotoxins (MC, CYN), neurotoxins (ANTX, STX), and skin irritants [16]. Several factors determine toxin production by cyanobacteria, including trophic, genetic, hydrological, environmental, and seasonal patterns [3,14]. A level of 100,000 cyanobacterial cells/mL (which is equivalent to approximately 50 mg chlorophyll-a/L if cyanobacteria dominate) is a guideline value for a moderate health alert in recreational waters [5]. *Aphanizomenon* spp. are known to produce a variety of cyanotoxins, including ANTX, CYN and STX [17,18,19]. *Aphanizomenon* spp. produce toxins in eutrophic conditions; studies suggest that nitrogen limitation may increase the extracellular release of toxin in CYN-producing cultures. The release of toxins through the cell membrane has been linked to NorM MATE proteins encoded by genes *cyrK* (for CYN toxins) [15,19,20] and *sxtM* (for STXs) [20,21]. Extracellular toxins are directly bioavailable and in direct contact with aquatic organisms and water users during bloom development and even after bloom dissipation. Some of these cyanotoxins have slow natural photo- and biodegradation (particularly CYN) [20] and need more monitoring for ecological and health risk assessments in waters commonly affected by *Aphanizomenon* spp.

#### 2.1.1. Anatoxin-a 

ANTX is a naturally occurring organophosphate. Toxicity of ANTX has been well documented since the 1960s [22]. It is an acute neurotoxin that has been shown to be toxigenic to both animals and humans. Uncertainty exists about the effects of ingestion of ANTX at low levels over extended periods of time [23]. The LD_50_ of ANTX is 200–250 µg/kg body weight in rats [24,25]. ANTX binds to nicotinic acetylcholine receptors where it mimics the natural ligand, acetylcholine [26], binding at 20 times higher affinity than acetylcholine. Normally, binding of acetylcholine to its receptor in neuronal membranes induces a conformation change in the receptor which results in the opening of an ion channel in the membrane, allowing calcium and/or sodium ions to move across the lipid bilayer [22]. This “depolarization” of the membrane allows for muscle contraction. Normally acetylcholinesterase quickly degrades acetylcholine, halting the activity. ANTX produces the same effect when it binds to the acetylcholine receptor, except that the binding is irreversible. After continuous stimulation of the neuromuscular junction a desensitization or “block” may follow, resulting in death due to respiratory paralysis [22]. Symptoms of ANTX ingestion include muscle tremors (involuntary muscle contractions or fasciculations) that can often be seen under the skin, followed by decreased movement, rigidity, exaggerated breathing, cyanosis, convulsions, respiratory paralysis and death [26]. Depending on the dose and species exposed, death can occur anywhere between a few minutes to 3 h. In some species ANTX exposure can cause significant effects on blood pressure, heart rate, and acidosis [27]. There are no human health standards for ANTX. The highest no-observed-adverse-effect level in mice (NOAEL) is 2.5 mg/kg-day [2]. While ANTX does not have a NOAEL for humans, researchers have developed a guideline value for ANTX in drinking water of 1 μg/L based on mice studies that would offer an adequate margin of safety for humans [23].

#### 2.1.2. Cylindrospermopsin

CYN is a naturally occurring liver toxin that is found typically in warm tropical waters but, has a presence worldwide. CYN is found in cyanobacteria such as *Cylindrospermopsis raciborskii* detected in Australia, Hungary [28], and the U.S. [29]; *Umezakia natans* in Japan [30]; *Anabaena bergii* in Israel [31]; *Raphidopsis curvata* in China [32]; and *Aphanizomenon ovalisporium* in Australia and Israel [31,33]. CYN is a potent inhibitor of protein synthesis [34]. In addition, studies have shown that this toxin is extremely stable. While CYN rapidly degrades in sunlight, it is resistant to degradation by temperature or pH [2]. P-450 metabolism is thought to be important in the toxicity of CYN. Studies by Runnegar et al. indicate that the toxicity of CYN may be due to the production of a CYN metabolite since toxicity in the presence of P-450 inhibitors is greatly reduced [35]. CYN has also recently been reported to be a suspect carcinogen, as it has been shown to be both mutagenic and cytotoxic [36]. The LD_50_ (oral) in mice was determined to be approximately 4–6 mg/kg [22]. The EPA also evaluated the health effects of CYN and derived an RfD in its 2015 ‘Health Effects Support Document for the Cyanobacterial Toxin Cylindrospermopsin’ [2]. The kidneys and liver appear to be the primary target organs for CYN toxicity. A critical study for the derivation of the CYN RfD was conducted by Humpage & Falconer (2003) based on drinking water exposure in mice [37]. The critical effect of cyanotoxins noted by the EPA was kidney damage, including increased kidney weight and decreased urinary protein [2].

#### 2.1.3. Microcystins 

MCs are a class of cyclic peptides, typically containing seven amino acids. Those found in freshwater are predominantly produced by species of *Microcystis, Planktothrix* (*Oscillatoria*)*, Anabaenopsis, Cylindrospermopsis and Aphanizomenon* [38]. This group of natural toxins include MCs and CYN [38]. MCs are hepatotoxins and once ingested, travel through the body to the liver, where they are stored. MC-LR is the most common MC and is an extremely acute toxin. The LD_50_ by the intraperitoneal route is approximately 25–150 µg/kg of body weight in mice; the oral LD_50_ (by gavage) is 5000 µg/kg of body weight in mice, and higher in rats [39]. With an estimated 100 MC congeners identified and named according to variable amino acids positions, complete evaluation of supplements or cyanobacterial material for all congeners is not feasible [40]. The provisional WHO guideline of 1 μg/L MC-LR is used if local health authorities suspect a risk to human health [4,5,16]. Because of repeated MC findings in BGAS, many countries have elected to develop regulatory limits for the amount of MC in these products, including businesses harvesting and selling AFA [41]. The EPA evaluated the health effects of MCs and derived a RfD in the 2015 document “Health Effects Support Document for the Cyanobacterial Toxin Microcystins”. The derivation of the MCs RfD was established by Heinze et al. in 1999, based on rat exposure to MC-LR in drinking water [40]. It is well known that MC-LR is the most toxic form of an estimated ~100 known congeners of MC [14,42]; therefore, the EPA established an RfD for MC-LR and used it as a surrogate value for the other ~100 MC congeners.

#### 2.1.4. Saxitoxin 

Another toxin produced by AFA is saxitoxin (STX), a paralytic shellfish poison (PSP) found in both marine and freshwater environments. The term saxitoxin can also refer to a class of more than 50 structurally related neurotoxins (known collectively as “saxitoxins” or STXs). Humans and other animals are commonly poisoned by STXs after they are taken up by filter-feeding bivalves and crustaceans, which ingest and concentrate the toxin within their tissues and organs [4]. STX-related poisonings are well known in Oregon, Washington, California, Alaska and many of the New England states [5,14]. Worldwide STXs have been documented in Australia, New Zealand, South Africa, China, Thailand, Japan and coastal areas of Western Europe. In recent years there appears to be an increase in STX intoxications. However, it is unknown whether this is a true increase in frequency, influenced by warming climatic patterns or simply a reflection of increased awareness or improved methods of detection. STXs exert their toxic effects by binding with high affinity to voltage-gated sodium channels. This binding blocks the passage of sodium ions across biological membranes resulting in a “blockade” of nerve signal transmission. STX is also capable of interfering with potassium and calcium-mediated ion channels. In the case of potassium channels, the STX effect is slightly different. Instead of blocking the potassium channel outright, it binds at the channel site, requiring stronger membrane depolarization to open the channel [43]. This results in an overall reduction of potassium conductance across membranes. STX acts on calcium channels in a manner similar to its action on sodium channels, although its ability to block calcium channels is not as efficient [43]. STX is fatal to humans even in very low doses. STX is also capable of entering the human body via a cut or other open wound, and the predicted human LD_50_ based on previous mice studies, via this route of exposure has been estimated to be 50 µg/kg body weight [5,14]. Currently the acute RfD, used in place of the NOAEL, is 0.5 μg STX equivalent/kg-day [44]. Humans develop a variety of symptoms, ranging from slight tingling or numbness of the face and extremities, to complete respiratory paralysis. Neurological symptoms present themselves shortly after ingestion [45]. 

### 2.2. Fresh Water

AFA is naturally present in freshwater sources [14]. CyanoHABs occur during optimal environmental conditions for cyanobacterial growth, including warm water (temperatures between 50–86°F), phosphorus concentrations greater than 30 μg/L, and high nitrogen content [4]. These conditions are often found during water pollution events, such as runoff from agricultural operations, or golf course or residential chemical pollution. During CyanoHABs many species of cyanobacteria produce toxins, which can be lethal or extremely harmful to both humans and animals, even at very low concentrations [46]. Cyanotoxins are toxic substances released by certain species of both marine and freshwater cyanobacteria during CyanoHABs, which are characterized by rapid, unchecked growth of cyanobacteria promoted by the presence or sudden addition of nutrients (especially nitrogen and phosphorus) into waters [14].

Throughout the literature AFA has demonstrated the ability to produce ATX, CYN, MC, and STXs under certain conditions and in certain geographical areas. Overall the *Aphanizomenon* spp. show evidence for moderate to high toxin production particularly with MCs, CYN (11–41% of total toxins, up to 58–63% under certain conditions), ATX (7–47% of total toxins), and STXs (7–35% of total toxins) [15,17]. While it is generally accepted that the *Aphanizomenon* spp. carry toxigenic genes and produce ATX, CYN, MCs and STXs in varying degrees, uncertainty exists over whether AFA has the genes for toxigenicity and if it does, whether it produces toxins in differing environmental conditions. In 2015, genome analysis by Šulcius et al. revealed the presence of non-coding sequences belonging to ANTX gene cluster that indicates AFA may have contained genes responsible for the production of cyanotoxins [47]. While the non-coding genes may have been present, the two AFA strains (2012/KM1/D3 and 2012/KM1/C4) that were isolated from bloom samples obtained from the Curonian Lagoon did not produce any cyanotoxins [47]. Although high concentrations of MC toxins were found in an oligo-mesotrophic lake in the Baltic Lake District, Germany, researchers discovered that the MCs were not produced by the dominant cyanobacteria present (*Dolichospermum circinale* and AFA) but by small numbers of *Microcystis cf. aeruginosa* and *Planktothrix rubescens* that co-habited the blooms sampled [48]. AFA was tested with HEPF/HEPR primers, but without any amplification for the *mcyE* gene responsible for MC toxin production [48]. Since AFA was responsible for 80% of the bloom’s total biomass [48], contamination with *Microcystis aeruginosa* is likely. This cyanobacterial co-occurrence also supports findings by Palus et al. in 2007, where cyanobacterial blooms were found to be most intense between August and September, producing the highest concentrations of MC toxins seen in the study [46]. *Microcystis aeruginosa* and AFA were the most dominant cyanobacteria present within the blooms during periods of high MC concentration [46], making determination of the cyanobacteria responsible for the MC production more difficult. 

More direct evidence for AFA toxigenicity was found in the following studies. In 2007 Fastner et al. detected measurable amounts of CYN toxin in 21 German lakes with high concentrations of *Aphanizomenon* spp. [28,49]. CYN was detected in 19 of the 21 lakes at concentrations ranging from 0.002–0.484 μg/L (phytoplankton + suspended particles) and 0.08–11.75 μg/L dissolved in water [28]. The maximum CYN measured in a total sample of water was 12.1 μg/L [28]. *Aphanizomenon gracile* is highly correlated with CYN concentrations in the lakes and was suspected to be the major producer of the detected CYN toxin [49], and may have led to potential contamination. In 2006 Preussel et al. identified three isolates of AFA from two German lakes, which were found to produce large amounts of CYN [17]. This was considered the first report of CYN in AFA strains [17]. CYN-synthesis of the strains was shown both by liquid chromatography-tandem mass spectrometry (LC-MS/MS) analysis and detection of PCR products of gene fragments [17]. The strains contained CYN in the range of 2.3–6.6 mg/g of cellular dry weight [17]. In 2007 Faster et al. also confirmed that AFA was a CYN-producing species, which often inhabits water bodies in temperate regions such as Germany and Portugal [17,28,50]. A risk assessment is recommended to confirm findings in geographic locations, suggesting that toxigenicity may vary by region [28]. *Aphanizomenon var klebahnii* was identified in 2009 in the Czech Republic as a potential producer of CYN toxins [51]. ANTX was also listed as a toxin produced by AFA, found in flamingos in Lakes Bogoria and Nakuru in Kenya. [1,52]. The birds were observed staggering and convulsing prior to death, and post-mortem studies found ANTX levels in the liver capable of causing death at 1.06–5.82 μg/L fresh weight [53]. In addition to ANTX, MC-LR was observed from 0.21 to 0.93 μg/L fresh weight within the bird livers post-mortem [53].

In addition to CYN and ANTX, there was evidence of STX when AFA was the dominant species in the Montargil Reservoir, Portugal [54]. To confirm the production of neurotoxins, a strain of AFA was isolated and established in culture and confirmed by high performance liquid chromatography with post column fluorescence derivatization HPLC-FLD [54]. A study in 1986 similarly found that AFA (NH-5) may produce neo-saxitoxin and saxitoxin, which was confirmed with thin-layer chromatography and HPLC [53]. A 2001 study by Ferreira et al. also detected STX toxins in cultures of AFA, isolated from the Crestuma-Lever Reservoir, using high performance liquid chromatography (HPLC) [53]. This finding was supported by claims from Liu et al. in 2006 that AFA has been reported in several countries to produce STXs and associated toxic effects [8]. Acute toxicity testing was performed by mouse bioassay using extracts from the lyophilized material and clear symptoms of STX intoxication were observed [8]. High performance liquid chromatography with post column fluorescence derivatization (HPLC-FLD) and liquid chromatographic mass spectrometry technique (LC-MS) analysis of extracts from cultured material demonstrated that STXs were produced by AFA blooms in China [8]. This was the first study reporting chemically and toxicologically confirmed STXs related to AFA in China [8]. Gkelis et al. identified AFA and *C. raciborskii* as potential producers of STX, while confirming *A. gracile* did, in fact, produce STX [55]. It was also noted that the STX gene cluster may vary biogeographically [55].

### 2.3. Dietary Supplements

Dietary supplements are largely self-regulated, although there are some safeguards in place following the passage of the 1994 Dietary Supplement Health and Education Act (DSHEA) by the United States Congress and its implementation by the Food and Drug Administration (FDA) [9]. In the United States, the FDA has determined that AFA is a dietary supplement; therefore, it is not subject to regulation as a drug, provided that the health benefits claimed by the manufacturer do not include the cure or treatment of a specific disease such as depression or cancer [41].

The harvesting of cyanobacteria for production as dietary supplements has recently come under scrutiny, as the production of these BGAS suffer from less strict quality controls than other food products or pharmaceuticals. In addition, BGAS are marketed internationally and sold widely over the counter and via the Internet. Although insufficient evidence exists, BGAS are reported to have beneficial health effects including weight loss, increasing alertness and energy, and mood elevation for people suffering from depression [2]. In children, they have been used as an alternative, natural therapy to treat attention deficit hyperactivity disorders (ADHD) [2]. Although AFA-based BGAS have generally been found to be non-toxic, the methods of cultivation in natural waters with inadequate quality controls allows for contamination by other toxigenic species. AFA is primarily harvested from Upper Klamath Lake in Oregon, USA where harvesting for supplement use started in the 1980’s. Other cyanobacteria species have been found to contaminate the products, since the AFA is harvested from the natural environment. As a potential consequence of contamination, the presence of microcystins (MCs) and alkaloid cyanotoxins have been found in BGAS [56]. Studies have found BGAS contaminated with MCs ranging from 1 μg/g up to 35 μg/g [57]. It is uncertain whether AFA or contamination by other species of cyanobacteria is the source of the MC toxins. 

For nearly 30 years, AFA has been harvested for blue-green algae supplements and constitutes a significant portion of the health food supplement industry in North America [41]. AFA is primarily harvested from cyanobacteria collected from Upper Klamath Lake in Oregon to produce BGAS [10,58]. In southern Oregon, growth of *Microcystis aeruginosa* is a regular occurrence together in blooms with AFA [10]. Because *M. aeruginosa* regularly coexists with AFA it can be collected inadvertently during the harvesting process, resulting in MC contamination of BGAS [10]. In 1996, in response to bloom advisories and BGAS consumer concern, the Oregon Health Authority (OHA, then known as Oregon Health Division) and the Oregon Department of Agriculture (ODA) established a regulatory limit of 1 μg/g for MCs in BGAS and tested BGAS products for the presence of MCs [10]. MCs were detected in 85 of 87 samples tested, with 63 samples (72%) containing concentrations greater than the regulatory limit of 1 μg/g. HPLC and ELISA toxin detection methods identified MC-LR as the predominant congener within the BGAS [10]. 

Saker et al. (2007) supports the findings that many AFA-based BGAS may be contaminated with MC-producing strains of *Microcystis* spp., and recommends that genetic testing be done to identify the organism responsible for MC production [59]. Previous studies of AFA samples have relied on the ELISA assay, which does not give information on the various chemical forms of MC [59]. Although the health benefits of BGAS are promoted, there are a growing number of studies showing the presence of toxins, some of which (for example MCs) are known to adversely affect human health [59]. In 2012, Heussner et al. analyzed 13 commercially available BGAS in Germany for the presence of cyanotoxins. All samples were analyzed and confirmed by PCR for the presence of the mcyE gene, a part of the MC and nodularin gene cluster [11]. Of all products tested, ten consisted of AFA, five of Spirulina platensis, and three of Chlorella pyrenoidosa [59]. *Spirulina* spp. are generally regarded as safe within the BGAS industry as of this time [11]. Only AFA BGAS products tested positive for MCs as well as the presence of the mcyE gene [11]. In 2015, a confirmed case of MC poisoning in a dog was documented following the consumption of a BGAS containing organic AFA daily for just 3 weeks [9]. While several brands of supplements of AFA have been found in the past to contain ANTX and its congeners [60,61], neither ANTX nor CYN were found in any of the supplements in the 2012 study. All products containing AFA tested positive for MCs at levels ≤ 1 μg MC-LR equivalents/g dry weight [11]. In 2017, Roy-Lachapell found that out of the 18 BGAS products analyzed, 8 contained cyanotoxins at levels exceeding the tolerable daily intake values [56]. Toxic strains of AFA may be found occasionally in some supplements, and have been reported to produce STX and ANTX toxins [56]. Some strains of AFA are known to produce STX and beta-methylamino-l-alanine (BMAA) [56]. Dietary supplements containing AFA had total MC toxin contents of between 0.8 and 8.2 μg/g [56].

## 3. Discussion

Cyanotoxin advisories and guideline values vary from state to state, and not all states have advisory and guideline values. To date, fifteen states have included recreational guideline values for cyanotoxins as part of their response protocol. The EPA currently lists Oregon’s options for recreational water guidance/action level as public health advisory over >100,000 cells/mL of “toxigenic species”, or >40,000 cells/mL of *Microcystis* or *Planktothrix*, and toxin testing for MC >10 μg/L, ANTX >20 μg/L, CYN >6 μg/L, and STX >100 μg/L [14]. Currently, for the purpose of issuing public health advisories, Oregon Health Authority excludes AFA from calculation of combined cell counts of toxigenic species. Other states include all *Aphanizomenon* spp. in their list of potentially toxic cyanobacteria, e.g., toxigenic taxa include *Anabaena, Microcystis*, *Planktothrix*, *Nostoc*, *Coelosphaerium*, *Anabaenopsis*, *Aphanizomenon*, *Gloeotrichia*, *Woronichinia*, *Oscillatoria*, *and Lyngbya* [4].

There is evidence to support *Aphanizomenon* spp. as a known toxin producer specifically for cyanotoxins: CYN (11–41%, up to 58–63% under certain conditions), ANTX (7–47%), and STXs (7–35%) [15]. In 2017, researchers Chernova et al. found evidence through PCR and LC-MS/MS of ANTX in Sestroretskij Razliv Lake, Russia, produced specifically by AFA [62]. Environmental conditions as well as bloom composition should be monitored closely due to the potential for changes in toxicity under increases in sunlight and temperature. Mariani et al. discovered that AFA and *Aphanocapsa* spp. dominated total CyanoHABs during periods of maximum MC concentration in Sardina, Italy [3]. There is still disagreement on whether AFA has the ability to produce cyanotoxins and this may be dependent upon environmental conditions and location. In 2012, a molecular study was carried out using 16S rRNA sequencing and results concluded that AFA did not show amplification for the toxin producing *mcyE* gene, and did not produce ANTX or STX [48]. Gkelis et al. also supported this theory of biogeographic toxicity stating that the *Aphanizomenon* spp. STX gene cluster may be biogeographically differentiated by county [55]. In Ireland AFA is commonly associated with blooms that have high MC concentration, but may be attributed to other contaminating species such as *M. aeruginosa* or *A. gracile* [63]. Researchers recommended further studies using molecular detection methods to determine whether AFA is a MC producer [63]. 

The first report of CYN in AFA was reported by Preussel et al. in Germany, 2006 through LC-MS/MS analysis and detection of PCR products of gene fragments [17]. In 2007, Fastner et al. also reported AFA as a CYN producer using microscopy and mass spectrometry identification methods [28]. CYN production was also reported in France and the Czech Republic where AFA was found to be a potential producer of CYN due to AFA dominated CyanoHABs where CYN was detected [51,64]. This was not confirmed with molecular detection methods and would be an area for future research. Ferreira et al. (2001) was the first report of STX toxins GTX4, GTX1, GTX3 [53]. This was followed in 2006 by Liu et al. who first reported STXs among AFA isolates discovered in China, through HPLC-FLD and LC-MS detection methods [8]. Among dietary supplements, the toxin-producing *mcyA* gene was detected in all AFA dietary supplements tested, which could be attributed to contamination by other toxic cyanobacteria such as *Microcystis* spp. [59]. Similarly, in 2012 Heussner et al. found all AFA products in their study tested positive for MCs and the *mcyE* gene [11]. 

The evidence for production of ANTX, CYN, MC, and STX, by AFA is of increasing concern, given that AFA occurs with high frequency in freshwater ecosystems throughout the U.S., and is regularly and increasingly used as a dietary supplement. Not only is AFA abundant in CyanoHAB events, but it appears to survive well in poor growth conditions as well, even at low temperatures [5]. AFA production of CYN and ANTX still remains uncertain [15]. Apart from AFA, the remaining seven *Aphanizomenon* spp. of this genus have only been described morphologically [15]. No phylogenetic data are available which can support their assignment to the genus *Aphanizomenon* and distinguish them as separate species [15]. AFA may have seasonality and/or biogeographical requirements for toxin production which should be established in order to confirm safety of both recreational water use and dietary supplements. 

One finding of this review is the large amount of uncertainty that exists over the potential for AFA to carry toxin genes and produce toxins. Several earlier studies have misidentified *Aphanizomenon* spp. for AFA and therefore their findings cannot be confirmed or used as evidence to support the presence of toxin production by AFA [32,65]. Without proper species identification, it is impossible to tell whether the cyanobacteria are AFA, or another species of *Aphanizomenon* such as *A. gracile*, which is a known toxin producer [15,66]. In addition, the data are currently insufficient to develop solid quantitative recreational values for cyanobacterial cell densities related to negative health outcomes [67]. Previous studies by Heinze et al. 1999 [40] and Humpage and Falconer in 2003 [37], have served as references for WHO, EPA and the Oregon Health Authority [58] to develop RfD for MCs and CYN toxins. No current NOAELs exist for ANTX or STX toxins, therefore TDI and guideline values cannot be properly assessed at this time. Because exposures can be acute (e.g., drinking water), sub-acute (e.g., recreational swimming exposure), and chronic (e.g., dietary supplement use), a single guideline value for any cyanotoxins is not appropriate [5]. Rather, it is recommended that a series of guideline values associated with incremental severity and probability of health effects should be defined at all three levels (acute, sub-acute, and chronic) [5].

## 4. Conclusions

Cyanobacteria blooms are commonly a mixture of toxic and non-toxic genotypes, and associated toxin concentrations can be highly variable both spatially and temporally [52,68]. Additionally, there is evidence that AFA carries toxin genes and produces significant levels of CYN and STX [17,28,50,51,69]. These toxins can potentially harm people, pets, livestock, aquatic animals, birds and other wildlife. In past analysis of AFA supplements, researchers have found the presence of a multitude of toxins including ANTX, STX, CYN, BMAA and MC [11]. MCs have been identified as the most common contaminants of BGAS [9,11]. Uncertainty remains regarding whether AFA was properly identified in earlier studies and was even reclassified to unidentified species of *Aphanizomenon* in two studies [32,65]. AFA-based BGAS may be contaminated with other more toxigenic species of *Aphanizomenon* or *M. aeruginosa* [10,41,59]. Overall, all studies looking more broadly at *Aphanizomenon* spp. have shown a moderate to high production of toxins. 

The presence of toxins in recreational waters and BGAS production environments remains a public health concern when CyanoHABs are present, with elevated levels of MCs being found and increasing reports of illness. Accumulated evidence from the peer-reviewed literature suggests that AFA carries toxigenic genes and has the potential to produce toxins under the right environmental conditions. Toxin production of cyanobacteria is highly variable, both within and between blooms and given the timing or seasonality of the bloom [52]. Evidence for production of ANTX, CYN, MC and STX by AFA is of immediate concern, given that AFA occurs with high frequency in freshwater ecosystems throughout the U.S. and is regularly and increasingly used as a dietary supplement. This issue should be evaluated regardless of the species of *Aphanizomenon* due to the potential for cross-contamination among species, and lack of regulation and testing among dietary supplements. Further investigation is warranted to improve our understanding of the effects that AFA dietary supplements have on consumers [57]. Surveys isolating individual colonies of *Microcystis* spp. from Upper Klamath Lake should be followed by laboratory cultivation and subsequent toxicological analyses to provide information on the toxicity of cyanobacterial contaminants [59]. 

The following recommendations are based on the most current global studies (2000–2017) to inform public health, regulatory and natural resource management agencies regarding AFA toxicity to make the most informed decisions. 

### 4.1. Recommendation to Post Educational Signs as a Precautionary Measure at First Sight of Visible Scum

These signs will help to educate and alert the public of the potential health risks associated with recreational water use during CyanoHABs. This step is particularly important for recreational users with small children or dogs. It is recommended that signs be posted at first visual confirmation of scum, while cell counts and/or toxicity analyses are underway. This action would reduce the risk of exposure to cyanotoxins.

### 4.2. Recommendation to Limit Harvesting of AFA to Months When Toxicity Is Lowest

The majority of studies indicate that cyanobacteria produce most toxins under environmental conditions, which are most favorable for their growth (warmer summer months). When temperature and light requirements are optimal for toxin production, AFA may produce more MCs than during times with less than optimal temperature and light [5]. This may lead to recommendations for seasonal harvesting of AFA when MC concentration would be lowest, and for increased testing and warnings for BGAS production and recreational use during times of high MC levels. This action would reduce global risk of exposure to humans and domestic animals from BGAS contamination with cyanotoxins.

### 4.3. Recommendation to Include AFA in Cell Counts during Visible Blooms

It has been demonstrated that AFA has the potential to produce toxins, and should be included in the cell counts of toxigenic cyanobacteria for public health advisories related to CyanoHABs. Ending exclusion of AFA from CyanoHAB advisory criteria would lead to increased frequency and duration of public health advisories and would reduce the risk of exposure to cyanotoxins. This may be evaluated and revised after additional evidence. 

### 4.4. Recommendation to Reduce Health Advisory Guideline Value for Cyanotoxin Levels

The EPA’s cyanotoxin guidelines were established under scientific rigor based on existing knowledge from published peer-reviewed scientific evidence on the adverse human health effects of toxins, criteria methodologies, and recreation-specific exposure parameters, reviewed by the Health and Ecological Criteria Division, Office of Science and Technology Office of Water, U.S. [14]. The health advisory guideline value for MCs should be reduced to match EPA’s proposed guidelines of 4 μg/ L [67]. Adoption of the EPA’s values as recreational use and swimming advisory values at the state level should be adopted under CWA 303(c) as ambient water quality standards. Addition of cyanotoxin water quality standards would eventually improve water quality and reduce exposure risks.

### 4.5. Recommendations for Proper Species Identification Using 16S rRNA Methods When Toxicity Levels Are Higher than Advisory Levels

Prior to 2007, several research studies had misidentified another toxin-producing cyanobacteria species for AFA. Previous attempts to differentiate toxigenic from non-toxic strains of the same species using microscopic methods have failed. Therefore, it is not recommended to identify species of cyanobacteria using only microscopic methods. During CyanoHABs events when cyanotoxin levels are above health advisory guideline values, advanced molecular methods such as 16S rRNA PCR should be used to determine specific strains of toxigenic cyanobacteria. This information would help to focus analysis for cyanotoxins and inform future evaluation of cyanobacteria toxigenicity.

### 4.6. Recommendations for Laboratory-Based Research to Confirm the Ability of AFA to Produce Toxins under Differing Environmental Conditions

Further research would provide an evidence base to improve recommendations for recreational and BGAS guidance values. It should be noted whether there is a visible algal bloom (include picture) at the time the sample is taken in addition to recording other environmental factors such as temperature (ambient and water), pH, and whether it is raining/snowing. Protocols for sample collection, preparation and storage should be evaluated for improvement. Both microscopic evaluation and ELISA testing should be done regardless of cell count. Cell count and ELISA results should be recorded and compared across time to note any predictive environmental factors for increased toxicity and to definitively prove whether AFA is a toxin producer. Molecular techniques should be employed to detect toxicity genes. This work will help to either confirm or refute the presence of toxin producing genes and better understand the risks associated with AFA in recreational waters and dietary supplements.

## 5. Materials and Methods

**Figure 1:**
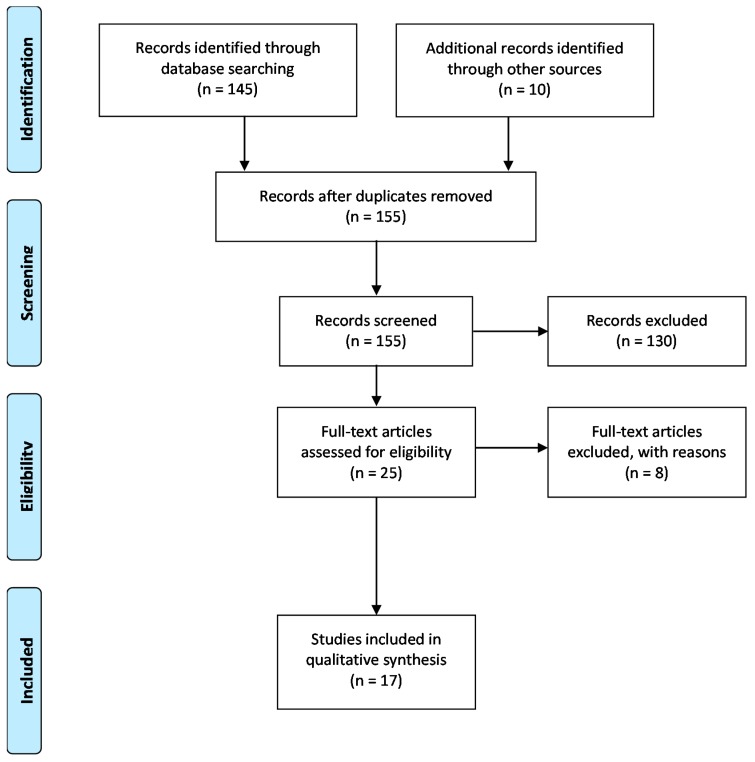
Article selection (PRISMA-P) flow diagram.

A systematic review of the peer-reviewed literature was conducted per the preferred reporting items for systematic review and meta-analysis protocol (PRISMA-P, Figure 1). The PRISMA-P is a 27-item checklist developed to strengthen the methodological quality, assessment of bias and quality of systematic reviews. No existing review protocols exist for this systematic review [70]. 

### 5.1. Search Strategy

A systematic search was conducted using the following electronic databases: NCBI’s PubMed, Web of Science, Cochrane CEN-TRAL. The following combinations of search terms were used: Toxicity and *Aphanizomenon flos-aquae*, cyanobacteria, harmful algal blooms, *Aphanizomenon* and toxicity in fresh waters, lakes, and dietary supplements. Two independent researchers screened titles and abstracts to identify eligible studies for inclusion within this systematic review. A literature search in NCBI’s PubMed database for ‘*Aphanizomenon flos aquae*’ returned 145 articles. Search term ‘Toxicity and *Aphanizomenon flos aquae*’ returned 50 articles. Ten more articles were identified using Science Direct through Oregon State University’s library; these mentioned AFA as being found in CyanoHABs but did not specifically describe AFA toxigenicity. Three additional review articles were identified through Cochrane. Articles which did not include measurement of toxin levels, or that did not specifically evaluate whether AFA was the producer of measured toxins, were excluded. In addition, systematic reviews were excluded in the analysis, but were considered for background information and article identification. Articles that included *Aphanizomenon* spp. were independently evaluated for relatedness. Titles with keywords “*Aphanizomenon*, *Aphanizomenon flos aquae*, toxicity including ATNX, CYN, MC, STX or PSP toxins, toxigenic, dietary supplements, and/or fresh-water” were included in the review. All relevant peer-reviewed articles up to 1 August 2017 were considered for inclusion.

### 5.2. Inclusion Criteria

Included studies were all peer-reviewed research articles measuring toxin levels and toxigenicity of AFA either in recreational waters, dietary supplements, or measuring toxicity and/or illness in either human cases or animal studies. Peer-reviewed articles and published books or reports with an ISBN number were included. All seventeen articles for inclusion listed in Table 1 referenced directly measured toxin levels associated with *Aphanizomenon* spp. dominated blooms or specifically measured genes and toxin production by AFA.

### 5.3. Exclusion Criteria

Articles were excluded if no English abstract was available. Review articles, student theses, newspapers or magazine reports, commentaries, correspondences, and letters were excluded from this review. Additionally, articles were excluded if there was no evaluation of toxin levels or direct human health outcomes due to exposure to AFA in either recreational waters or dietary supplements. Although two articles were found to be relevant to the search terms, it was later discovered there was a re-classification of the cyanobacteria from AFA to *Aphanizomenon* spp. These are considered for background and discussion but were excluded from the systematic review. 

### 5.4. Study Quality Assessment

For each included study, the quality of evidence was assessed using the GRADE (Grading of Recommendations: Assessment, Development and Evaluation) method. The GRADE method rates the quality of evidence presented in research studies for evaluation. Evidence is assessed based on five factors: risk of bias, imprecisions, inconsistency, indirectness of evidence and publication bias. The quality of evidence is rated on the following scale: “High Quality”, “Moderate Quality”, “Low Quality” and “Very Low Quality.” Randomized control trials are considered “High Quality” evidence but, can be downgraded if there is a serious risk of bias in the study. Additionally, observation studies begin as “Low Quality” evidence and can be upgraded due to large effect size (Relative Risk or Odds Ratio > 2), presence of a dose response or the presence of confounders against bias. The methodology is widely used and accepted by numerous organizations including the WHO. Articles were assessed, and tables were produced using the online GRADEpro toolkit, summary of findings table.

## Figures and Tables

**Table 1:** Characteristics of studies included in the systematic review for AFA toxigenicity (2000–2017).

Lead Author (Year)	Location	Findings	Method of Detection	Conclusions
Roy-Lachapelle (2017)	Canada	Out of 18 products tested, 8 contained cyanotoxins at levels exceeding WHO’s TDI. Supplements containing AFA had MC concentrations between 0.8 and 8.2 μg/g. Low amounts of BMAA (neurotoxin) were also found.	Lemieux and Adda oxidation. Chemical derivatization, laser diode thermal desorption, and liquid chromatography.	Some dietary products could be harmful upon long-term consumption due to the presence of cyanotoxins. A critical need exists for better monitoring for all BGAS, and guidelines for maximum intake.
Chernova (2017)	Russia	*Aphanizomenon flos-aquae* found to produce ANTX in Sestroretskij Razliv Lake	PCR and LC-MS/MS and Restriction Fragment Length Polymorphism (RFLP) analysis	Both dominant species *Aphanizomenon flos-aquae* and *Dolichospermum planctonicum* are ANTX producers.
Cires (2016)	Global	*Aphanizomenon* spp. known toxin producer specifically: CYN (11–41% of total toxins, up to 58–63% under certain conditions), ANTX (7–47% of total toxins), and SXTs (7–35% of total toxins)	Literature Review.	Although *Aphanizomenon* spp. are known toxin producers, the toxigenicity of AFA is still uncertain.
Mariani (2015)	Sardinia	AFA and *Aphanocapsa* spp. dominated total cyanobacteria.	ELISA, Mass spectrometer.	Species composition during periods of maximum MC concentration differed from typical in other Mediterranean sites.
Sulcius (2015)	Lithuania/Russia	Concentrations of cyanotoxins in scum materials increased from ~30~300 fold compared to bloom samples. AFA comprised ~19% of total cyanobacteria biomass. The most common toxin-producing cyanobacteria from Curonian Lagoon belong to the genera of *Aphanizomenon* spp., *Microcystis*, and *Planktothrix*.	Microscopic, and chemical. Extraction of saxitoxins with 4 mM ammonium formate buffer and acetonitrile 2:3 ratio, Mass spectrometer, information dependent acquisition mode, and multiple reaction monitoring.	Larger concentrations of cyanotoxins were found in scum compared to blooms.
Dadheech (2014)	Germany	Although AFA dominated total phytoplankton at >80% contribution to total biomass, AFA did not show amplification for the *mcyE* gene, or STX and ANTX production.	Molecular analysis:16S rRNA sequencing, BLAST identification.	Differences seen in dominant taxon of field sample from *Dolichospermum circinale* in 2011 to AFA in 2012, with reduction in total MC content seen from 27.32 μg/L to 4.25 μg/L.
Gkelis (2014)	Greece	*C. raciborskii* and AFA are *potential* STX producers. *A. gracile* confirmed STX producer. MC: 3.9–108 μg/L, CYNs: 0.3–2.8 μg/L, and STXs: 0.4–1.2 μg/L *Aphanizomenon* spp. STX gene cluster may be biogeographically differentiated by county.	Microscopy, molecular, and immunologic methods: ELISA.	AFA was not found to be the dominant species in blooms, or a producer of toxins. Co-occurrence of more than one cyanotoxins in sites used for drinking water, agriculture, or recreation represent potential health risks.
Heussner (2012)	Germany	All AFA products tested positive for MCs and the *mcyE* gene. The contamination levels of the MC-positive samples were ≤1 μg MC-LR equivalents per g dry weight.	Colorimetric protein phosphatase inhibition assay (cPPIA), Adda-ELISA, Cell Culture, Liquid chromatography tandem mass spectrometry (LC-MS/MS), DNA extraction and PCR.	Recommendation for prohibition of marketing and sale of AFA-based dietary supplements in order to prevent acute and chronic exposure to MCs.
Mooney (2011)	Ireland	AFA, *Gomaphosphaeria* spp. and *Microcystis aeruginosa* were the most dominant cyanobacterial species associated with high MC concentration. AFA was dominant in 1/14 sites with lake area of 382 km^2^, and MC concentration of 1652 ng/μg Chl*a*	Synoptic survey of 14 sites, used high performance liquid chromatography-tandem mass spectrometry (HPLC-MS/MS).	Further studies are recommended to use molecular detection methods to determine whether AFA is a MC producer. It is unknown which species produced the toxins, only recorded dominant cyanobacteria and total toxin per area.
Blahova (2009)	France	CYN was found at 3 localities with *Aphanizomenon* spp. sub-dominated water blooms. Concentrations determined by ELISA (0.4–4 μg/L) were systematically higher than concentrations determined by LC/MS (0.01–0.3 μg/L).	ELISA and LC/MS.	AFA is a potential producer of CYN.
Brient (2009)	Czech Republic	*AFA var klebahnii* found to be a potential producer of CYN. Intracellular concentrations of CYN ranged between 1.55 and 1.95 μg/L.	LC-MS/MS.	AFA is a potential producer of CYN.
Palus (2007)	Poland	AFA dominated blooms August–October. The concentration of MC in water did not exceed 1 μg/L, Cyanobacteria co-occurrence found with *E. coli*.	Protein phosphatase inhibition assay (PPIA), ELISA and HPLC.	Phytoplankton biomass and genotoxicity of CyanoHABs should be assessed to avoid public health issues.
Fastner (2007)	Germany	Concentrations reached up to 73.2 μg CYN/g dry weight. Study confirmed AFA is a CYN-producing species frequently inhabiting water bodies in temperate climatic regions.	Microscopy, Mass-spectrometer.	*Aphanizomenon* spp. may be an important CYN toxin producer in Germany waters. A world hazard analysis and risk assessment is recommended to confirm by geographic regions.
Saker (2007)	Australia and Canada	*mcyA* gene was detected in all 12 AFA dietary supplements, suggesting contamination by *Microcystis* spp.	Multiplex PCR.	Dietary supplements containing AFA are more at risk for contamination by *Microcystis* spp., and should be monitored. Laboratory and toxicological analysis of Upper Klamath Lake cyanobacteria would provide useful information to inform and protect human health.
Preussel (2006)	Germany	Toxin CYN detected in the range of 2.3–6.6 mg/g of cellular dry weight.	LC-MS/MS analysis and detection of PCR products of gene fragments.	First report of CYN in AFA strains.
Ferreira (2001)	Portugal	Presence of PSP toxins: GTX4, GTX1, GTX3, and Cs toxin present either in cells of AFA or in other toxic isolates.	High performance liquid chromatography (HPLC) using 2 isocratic elution systems.	AFA known STX producer, but *A. circinalis* is also found to be potential STX producer. More work is needed to understand the toxicological profiles of cyanobacteria.
Liu (2006)	China	STXs produced by AFA bloom. Significant glutathione-S-transferase (GST) and lactate dehydrogenase (LDH) increases, together with decrease of the glutathione (GSH) level, were measured.	High performance liquid chromatography with post-column fluorescence derivatization (HPLC-FLD) and liquid chromatographic mass spectrometry technique (LC-MS).	The results indicate a potential role of STXs intoxicating and metabolizing in test animals.
